# Comparison of Time Resolved Optical Turbidity Measurements for Water Monitoring to Standard Real-Time Techniques

**DOI:** 10.3390/s21093136

**Published:** 2021-04-30

**Authors:** Anne Pallarès, Philippe Schmitt, Wilfried Uhring

**Affiliations:** 1 Laboratoire ICube—UMR 7357, Université de Strasbourg/CNRS, 2 Rue Boussingault, 67000 Strasbourg, France; pschmitt@unistra.fr (P.S.); wilfried.uhring@unistra.fr (W.U.); 2 Université de Haute Alsace, 2 Rue des Frères Lumière, 68100 Mulhouse, France

**Keywords:** turbidity, optical turbidity, acoustic backscattering, monitoring, suspended solids, optical tomography, time resolved, time-correlated single photon counting (TCSPC), water

## Abstract

Environmental water monitoring requires the estimation of the suspended solids load. In this paper, we compare the concentration range accessible through three different techniques: optical turbidity, acoustic backscattering and the newly in-lab developed time resolved optical turbidity. We focus on their comparison on measurements made in the laboratory on water suspensions of known particles and concentrations. We used laboratory grade kieselguhr, wheat starch and kaolin as suspended solid surrogates. The explored concentration domains are the ones, for the total suspended solid load, commonly encountered in wastewater and rivers in standard (less than 1 g/L to a few g/L) or extreme conditions such as floods or storm events (up to several dozen g/L). Regarding the operable concentration domain, the time resolved optical turbidity shows a clear advantage upon the other methods, whatever the kind of particle is.

## 1. Introduction

Water is a precious good. Water quality monitoring strongly contributes to Goal 6 of the UNESCO Sustainable Development Goals (SDG) project [1], which aims, among other targets, to halve the proportion of the world’s population without sustainable access to safe drinking water supply and sanitation. Clean water is also an indispensable foundation for many other SDGs. Laws worldwide tend to preserve it. In Europe, the Water Framework Directive [2] rules impose regular water quality estimation. Particular attention is given to the total suspended solids (TSS) load as well in rivers as in wastewaters. The aim is to monitor the sediment transport that potentially carries pollutants and causes occlusion [3].

Thus, optimal water management involves at least a periodic estimation of the TSS concentration. Even if time consuming, costly and of low periodicity, water sampling followed by its laboratory analysis remains the most deployed method [4]. Regarding control and regulation, the major disadvantage of this method is the considerable period of time (hours, days) between the sampling and the results of the analysis. This operation also has multiple disadvantages such as the low representativeness of the sample sucked by the sampler or the possible chemical or physical evolution of the sample from its sampling until its analysis.

The interest of real-time measurement of TSS concentration is to minimize or even replace sampling. Unlike samples, it provides continuous information giving access to a possible real-time management of a wastewater network or a treatment plant. A recent inventory of the different real-time TSS monitoring techniques made by Rai and Kumar [5] shows that optical turbidity remains the most frequently used technique. A bijective relationship, mostly considered as linear, is expected between particle concentration and optical turbidity [6,7]. Unfortunately, it is often omitted that this relationship requires stable shape and size of particle distribution [8]. In addition, optical turbidity has a few other drawbacks. Voichik et al. [9] brought back to light the known dysfunction of optical turbidimeters at high concentrations. In addition, the work of Rymszewicz et al. [10] shows a great disparity in the response of optical turbidimeters regardless of their operating principle. For this paper, we used a commercial turbidimeter and expected a linear relation between particle concentration and the measured turbidity.

To monitor TSS concentrations, a more confidential but powerful method, particularly due to the complexity of the signal analysis, is acoustic backscattering [11,12]. Such data can be obtained from different types of devices, from Acoustic Doppler Current Profilers (ADCPs), Acoustic Doppler Velocity Profilers (ADVPs) to simple Acoustic Doppler Velocimeters (ADVs) [13]. We used a commercial ADVP to record the acoustic backscattered amplitude, directly proportional to the acoustic turbidity. The simplest signal approach that does not need laborious instrument characterization nor complicated data inversion was applied. The acoustic signal is declined in two parts: attenuation due to particles and backscattering due to particles. A linear behavior is expected for the two dependencies.

The aim of this paper is to compare the performance of two well-known techniques, optical turbidity and acoustic backscattering, to a new instrumental method based on time-correlated single photon counting (TCSPC) used to measure the optical turbidity of water-based suspensions. Time resolved optical turbidity or TROT is the application of TCSPC to turbidity measurement. This technique, still of very low technology readiness level, is derived from previous studies on medical applications [14]. Very few applications of turbidity measurements through laser illumination are present in the literature.

Prerana et al. [15] proposed a first approach in 2012, on an exploratory basis, with the use of optical fibers and the measurement of light power returned through different samples of alkaline suspensions. The diffusion and absorption characteristics of the medium were made accessible through the measurement of the total interaction coefficient.

A first attempt to exploit time-correlated single photon counting (TCSPC) dedicated to drinking water was carried out by Wang et al. [16,17] in 2015 on formazin solutions of low and medium concentrations. Their work shows, at low concentrations, a linear dependence between the observed normalized number of photons and the turbidity of the sample. Their measurements were made on small samples, whereas our measurements were made directly in the medium.

Even if known since the 70′s [18], TCSPC has limited applications due to the need of adapted light sources and fast electronics. The laboratory work of Wang et al. is the only published research on water turbidity measurements by TCSPC. With the progress and needs in telecommunications, thus on ultrafast electronics and optics, TCSPC will be able to disseminate over various applications [19]. The use of laser diode and self-developed electronics, as proposed in this article, is a first step to a wider purpose of this technique. We have already compared the performances of TCSPC to standard turbidity measurement techniques on a large particle concentration domain.

We made laboratory measurements to check the behavior of the different techniques on known particles and over a large range of concentrations. Typical variations for the TSS concentration for wastewaters or rivers, are between 0.1 and 2 g/L during dry weather conditions. Alternatively, concentrations of more than 50 g/L have been observed during river floods and are more likely during rainy periods. As recalled in [9], conventional optical turbidity is unfortunately ineffective at high concentrations as the latter are encountered in exceptional periods such as river floods or storm weather. The need of an instrumentation covering the entire concentration range is thus clear and might be the instrumentation developed for TROT.

## 2. Materials and Methods

**Figure 1:**
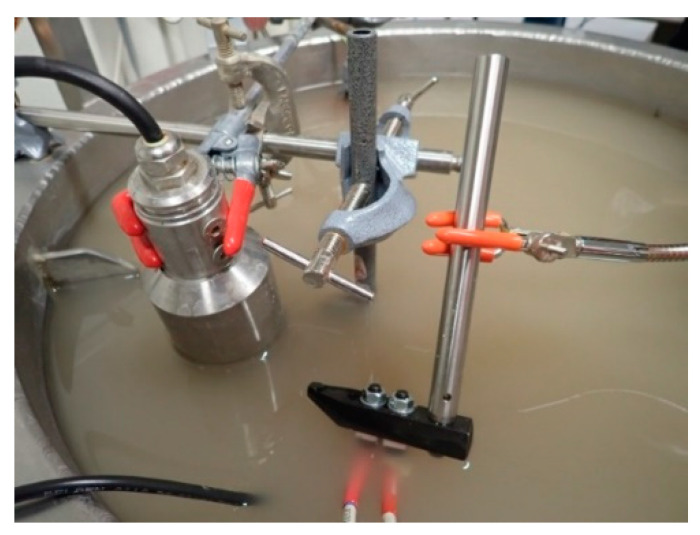
Picture of the experimental set-up.

All the types of measurements (optical turbidity, acoustic backscattering and TROT) were simultaneously performed in a 50 L tank filled with faucet water at room temperature and with various concentrations of particles. As shown in Figure 1, all the instruments are fixed on the tank walls and immersed. The tank was coated with a black blanket and the room darkened during measurements to avoid background light for both optical turbidity and TROT. At the bottom of the tank, a propeller ensures a homogeneous suspension.

We used kieselguhr and kaolin as mineral substitutes of TSS. The kieselguhr particles (Celite 500, Sigma-Aldrich, St. Quentin Fallavier, France) are of various shapes with a mean diameter of 18 µm and are representative of the mineral particle fraction in rivers and wastewaters. Kaolin powder (Sigma-Aldrich), with an ovoid shape of mean diameter 5 µm, corresponds to the fine mineral particle type. Wheat starch (S5127—Starch wheat, Sigma Aldrich) is a good surrogate for the organic fraction [20]. It is of ovoid shape with a mean diameter of 18 µm as for kieselguhr particles.

We investigated a large concentration range from concentrations below 0.1 g/L to several dozens of grams per liter, which covers common concentrations and reach the optical turbidimeter saturation zone.

### 2.1. Optical Turbidity

The optical turbidity was continuously recorded using a commercial Solitax Sc turbidimeter (Hach, Lognes, France). Its measurement technique is based on an infrared duo-scattered light technique. It combines nephelometry, measurement of diffuse radiation, with turbidimetry, measurement of the attenuation of a radiant flux. It was calibrated with 800 NTU formazin Standard. The manufacturer claims a measured TSS concentration domain between 0.01 and 50 g/L if well calibrated.

As illustrated in Equation (1), for a given particle concentration value *M*, we averaged the optical turbidity values *T* over the recording time, which was approximately 5 min. At least at low concentration, a linear dependence between particle concentration and turbidity is expected. A linear regression to obtain the values of the intercept *b* and slope *a* is performed on the turbidity data according to:

*T* = *aM* + *b* (1)

### 2.2. Acoustic Measurements

A UB-Lab system (Ubertone, Schiltigheim, France) with a 10 MHz central frequency transducer was used to perform acoustic measurements at 7.5 MHz. This frequency is a compromise between the signal efficiency for the size of the particles and the beam attenuation in the medium [21].

The backscattered acoustical signal was collected over the whole ultrasonic beam. However, it was interpreted only over the range of cells situated at more than three times the near field distance to avoid signal distortion.

As described in previous papers [22], we used the reference backscattering equation given in Equation (2). *V_rms_* is the averaged value of the root mean square voltage over many backscattered receptions and *r* the distance from the emitter to the measurement volume:

(2) $$ln\left(r\;V_{rms}\right)=ln\left(\frac{k_{p}k_{t}}{\psi }\right)+\frac{1}{2}lnM-2r\left(\alpha_{w}+\frac{3\chi_{m}M}{4\rho_{p}\langle a_{p}\rangle }\right)$$

Thus:

(3) $$ln\left(r\;V_{rms}\right)=\gamma -\delta r$$

The signification of the other parameters of equation Equation (2) can be found in Table 1. For a given frequency, they are instrument or particle-dependent constants. For each particle concentration *M*, we did a linear regression according to Equation (3) to obtain the values of the intercept *γ* and the slope *δ*. We kept only significant data: the acceptance criterion was a correlation coefficient verifying *r*^2^ > 0.85.

In a second step, we did another linear fit on the intercept and the slope values as a function of the particle concentration. For the intercept *γ*, a linear dependence with the logarithm of the concentration is expected, as shown in Equation (2). For the slope *δ*, a linear dependence with the particle concentration is foreseen by Equation (2). *α_w_* can be extrapolated from empirical formulas [23].

### 2.3. Time Resolved Optical Turbidity

**Figure 2:**
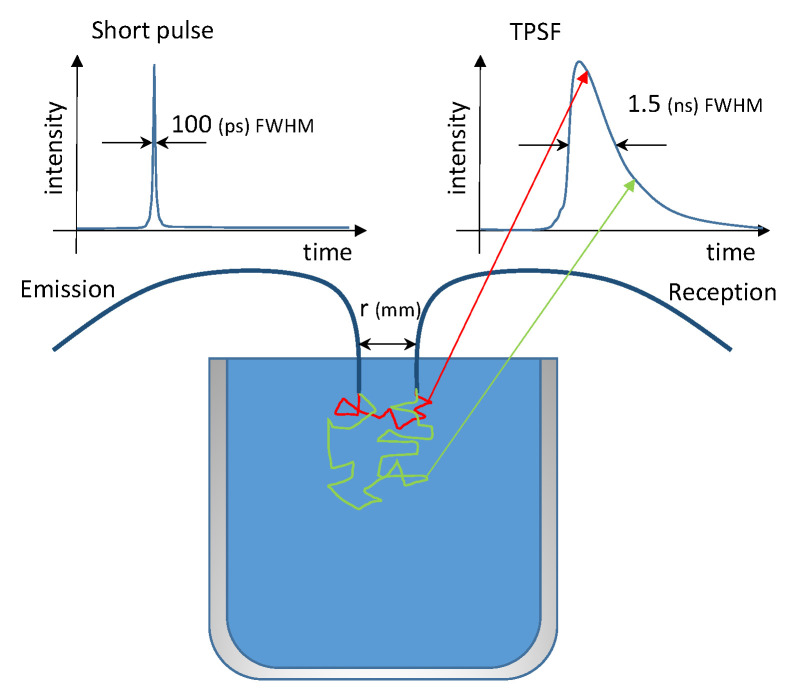
Principle of Time Resolved Optical Turbidity (TROT).

The principle of time resolved optical turbidity (TROT) is described in Figure 2. Periodically, a short pulse of photons, with a full width at half maximum (FWHM) of typically less than 100 ps, is injected into the turbid medium through the emission optical fiber. A second optical fiber, called the reception fiber, is placed beside the first one at a given distance *r*, which is typically a few tens of mm. Most of the injected photons are scattered and absorbed in the medium, some of them reach the surface of the medium and vanish and finally, some of them are scattered up to the reception fiber. Photons which follow the short optical path (red path) in the medium reach the reception fiber before the photons which follow the longer optical path (green path) leading to a different time of flight. When a photon is detected, the time of the corresponding detector pulse in the signal period is measured. The number of events with the same detection time builds up the waveform. As this process is stochastic, the formal short optical emission pulse is spread by the scattering effect of the turbid medium and we finally observe a temporal point spread function (TPSF) which lasts for a few nanoseconds.

Theoretically, as can be seen in Equation (4), the number of photons collected by the output fiber as a function of time and distance follows the diffusion equation in infinite medium so called Greens equation, known to be valid in biological samples [24]:

(4) Φ(r→,t)=(4πκt)−32exp(−μact−r24κt)

where the diffusion coefficient *κ* is given by:

(5) $$\kappa =\frac{c}{3\left(\mu_{a}+{\mu^{\prime }}_{s}\right)}$$

In Equation (4), *c* is the speed of light in the medium, *r* the distance between the emission and reception fiber and *t* the time elapsed since the pulse emission. The constants *µ_a_* and ${\mu^{\prime }}_{s}$ are, respectively, the absorption and the reduced diffusion coefficients of the medium. A relation between the number of collected photons, the shape of the curve (*µ_a_* and ${\mu^{\prime }}_{s}$) and the concentration of particles is expected. For each particle concentration, the histogram of TPSF was obtained over an acquisition time of 100 µs.

**Figure 3:**
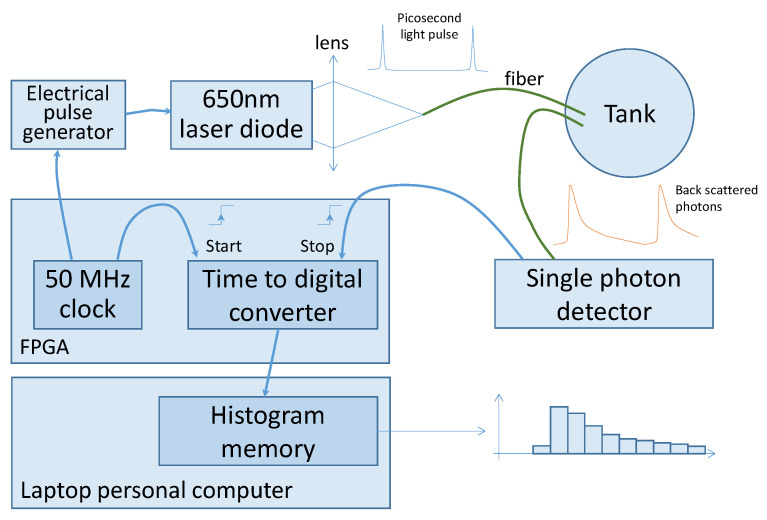
Schematic set-up of TROT.

Figure 3 shows our experimental TROT set-up. It consists of an in-lab developed time correlated photon counting system. A field-programmable gate array (FPGA) generates a trigger signal to a fast pulse generator [25], which drives a laser diode emitting a 100 ps FWHM pulse of light at a wavelength of 650 nm. At the set repetition rate of 50 MHz, i.e., a period of 20 ns, the mean optical power is less than 1 mW. The light pulse is brought to the tank via a 1 mm step index optical fiber. Another similar fiber brings the light back to a single photon detector, a single photon avalanche diode (SPAD), id100-MMF50 from ID Quantique, Carouge, Switzerland). The tips of the fibers mounted in parallel were fixed on a plastic support and submerged under the surface of the medium. The distance between the two fibers was *r* = 13.5 mm. The signal delivered by the single photon detector is time correlated to the trigger of the laser pulse generator due to a time to digital converter (TDC) synthesized in the FPGA [26]. The TDC converts the time between the laser pulse and the measured photon into a digital value with a quantum resolution of 100 ps.

All these components are compact; their integration, including the SPAD, in a reduced volume could be possible. That makes the new TROT approach compatible with the field operation which was previously inconceivable without progress in electronics and optics. Moreover, the probe, i.e., the part to be immerged in the water, could only consist of the two optical fibers. Consequently, all the electronics could be placed outside the water releasing the design constraints of the probe. In addition, silica core polyimide-coated fibers are ideally suited to harsh chemical environments and show low sensitivity to other aggressions such as electromagnetic perturbation.

The use of a time resolved single photon counting system should also provide a high dynamic range as well as a high sensitivity.

## 3. Results

As mentioned in Section 2, we used three different kinds of particles. Kieselguhr was used to demonstrate the feasibility of the TROT technique and to roughly compare it to optical and acoustic turbidity on a standard concentration domain. Higher quantities of kaolin and wheat starch were used to see how both acoustic backscattering and the TROT technique would react in the known dysfunction range of optical turbidimeters.

### 3.1. Optical Turbidity

**Figure 4:**
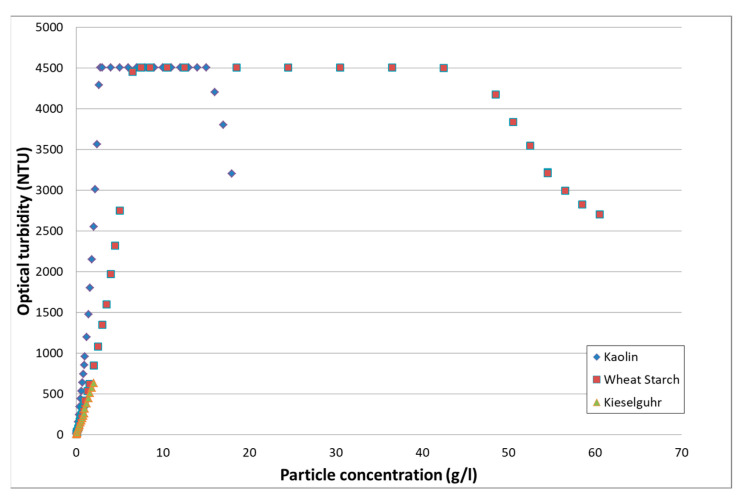
Evolution of the optical turbidity measured by a commercial turbidimeter as a function of the particle type and concentration.

The evolution of the optical turbidity measured by the commercial turbidimeter as a function of particle type and concentration is given in Figure 4. The green triangle curve represents the kieselguhr, on a concentration domain on which linear behavior is expected. The two other curves, red square, and blue lozenge are respectively for wheat starch and kaolin, on a larger concentration range, which includes optical turbidimeter dysfunction.

For a given particle type, the optical turbidity measured by the Solitax Sc turbidimeter shows a linear dependence with the particle concentration for common values encountered in waters. When increasing the particle quantity, as was performed for kaolin and wheat starch, the measured optical turbidity first reaches a plateau. This plateau will last over a concentration domain depending on the instrument and the particle type. Then, the measured optical turbidity decreases with increasing concentration. Thus, the same optical turbidity value corresponds to two different quantities of particles, which is a severe ambiguity drawback [27].

On the commercial optical turbidimeter operating zone, for concentrations between 0 and 2 g/L for all particles, we made a linear regression analysis of the optical turbidity according to *T = a M + b*. The results of the linear regression analysis are given in Table 2.

The values of *a* follow the expected optical turbidity behavior which is known to be inversely proportional to the particle diameter [8].

Therefore, a linear relationship between optical turbidity and particle concentration exists for very low concentrations of up to a few grams per liter, depending on the particle type. Consequently, optical turbidity is well adapted for water survey in standard dry weather conditions.

### 3.2. Acoustic Measurements

The acoustic turbidity measurements at 7.5 MHz are significant only for particle concentrations over 0.1 g/L. Under this value, the backscattered acoustic signal is not strong enough to give an acceptable linear fit of the data and is rejected.

#### 3.2.1. Attenuation

**Figure 5:**
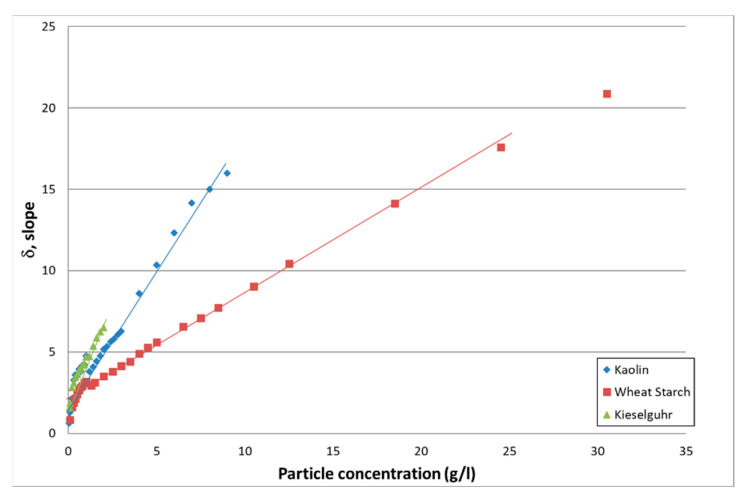
Evolution of the slope as a function of the particle type and concentration.

The value of the slope δ is, according to Equation (2) and Equation (3), directly linked to attenuation properties of the particles present in the flow. A linear dependence of the slope value versus the particle concentration *M* is expected. Figure 5 shows the plot of the slope value δ as a function of the particle type and concentration *M*. A linear regression analysis was carried out on the slope value according to *δ = a_s_ M + b_s_.* Results are presented in Table 3.

As can be seen in Figure 5, particularly for kaolin, the value of the slope, thus the intensity of the acoustic signal, slightly decreases due to excessive attenuation at high concentration. However, for all particles, the linear regression analysis was performed on the entire concentration domain.

As expected by theory and reflected by the value of *a_s_*, mineral particles, such as kieselguhr and kaolin, lead to more attenuation than organic particles such as wheat starch.

The results given in Table 3 show that a linear dependence of acoustic attenuation with particle concentration is expected over a large concentration domain. However, Table 3 shows that acoustics are deficient for low particle concentration (<0.1 g/L). On the other side, for a given particle, the linear dependence can be expected for concentrations well above the dysfunction zone of the optical turbidimeter.

#### 3.2.2. Backscattering

The intercept γ of the linear fit of the acoustic signal directly reflects the backscattering properties of the particles present in the flow. By performing a linear regression on γ versus ln M, a slope of 0.5 is expected by theory according to Equation (2).

**Figure 6:**
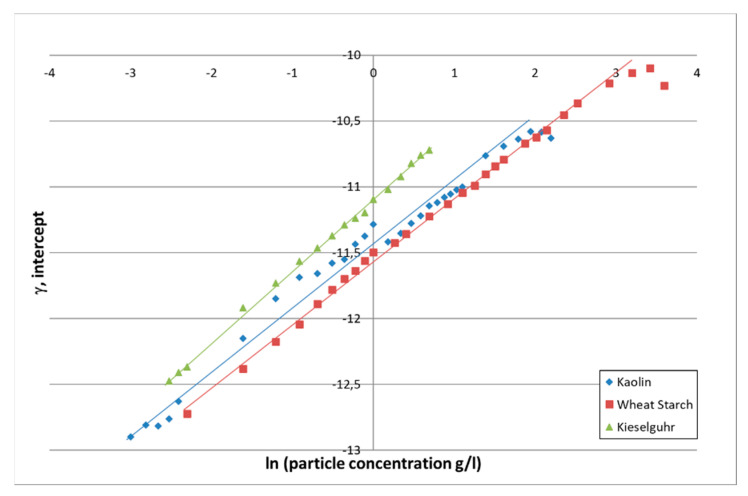
Evolution of the intercept value as a function of the logarithm of the particle concentration.

The behavior of the intercept as a function of the particle type and concentration is given in Figure 6. Again, a linear regression of the intercept accordingly to γ = a_i_ ln M + b_i_ was carried out on the data. The results are given in Table 4.

For all particles, the value of *a_i_* is close to 0.5 as expected by theory. As already observed on the attenuation and visible on the respective values of *b_i_*, kieselguhr has stronger scattering properties than kaolin. Also, as expected, organic particles such as wheat starch are less rigid and will cause less scattering.

Specifically, on the dependence of intercept on concentration, one can note the unsteady behavior in the case of kaolin. This is due to its small size for which the acoustic signal intensity is poor because of maladjusted frequency. When the particle size decreases, higher frequencies should be used to obtain sufficient backscattering signal intensity.

As in Table 3 and Table 4, the intercept shows a linear dependence of acoustic backscattering over the same concentration domain as seen for attenuation. A notable point is that we showed that acoustic data still operates for particle concentrations for which optical turbidity stops regular working.

### 3.3. Time Resolved Optical Turbidity

**Figure 7:**
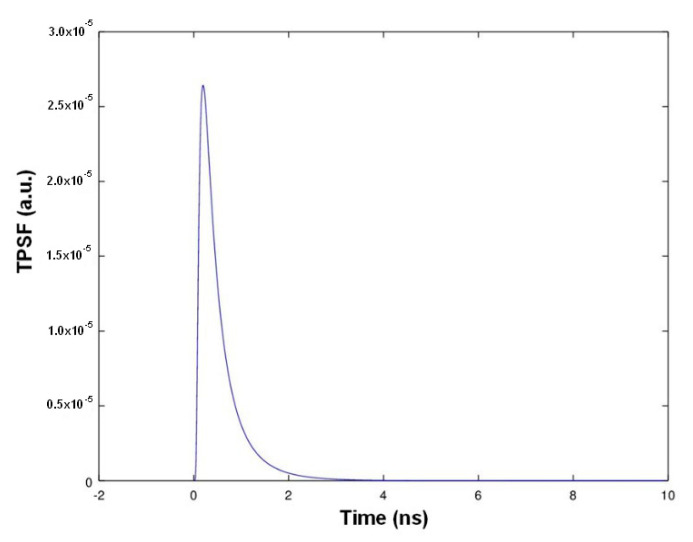
Theoretical number of collected photons as a function of time.

The TROT waveform is given by the temporal point spread function of the emission pulse. A shown in [24], the TPSF will depend on the absorption and diffusion properties of the illuminated medium. Given Equation (4), we plotted in Figure 7 the normalized Green function for an infinite medium defined by its refractive index *n* = 1.33, pure water, *µ_a_* = 0.005 mm^−1^, *µ_s_*’ = 0.6 mm at a distance of *r* = 13 mm from the source [28]. The number of photons collected by our TROT system is supposed to have the same shape as the TPSF shown Figure 7.

**Figure 8:**
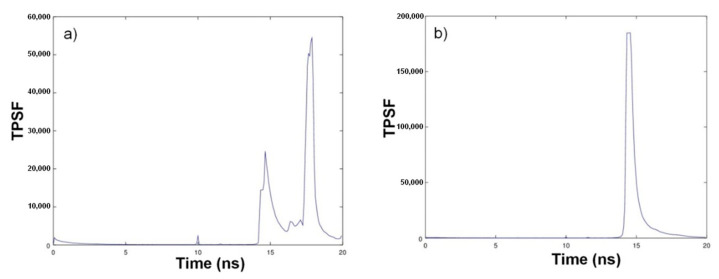
Number of collected photons as a function of time (**a**) @ 0.01 g/L; (**b**) @ 0.3 g/L.

The TPSF is function of the medium thus of the particle concentration. Its simplest observable parameter is the total number of photons. Figure 8 shows typical TPSF recordings obtained for kieselguhr suspensions.

For a low concentration of kieselguhr (0.01 g/L), one can see two distinct parts in the temporal profile (Figure 8a). The first part, the peak at the left, is the real signal returned from the medium. The second peak appearing 3 ns later corresponds to the photons reflected by the metallic walls of the tank. At higher concentrations, at 0.3 g/L (Figure 8b), the reflection peak is drowned in the signal. Thus, at low concentrations, the time-resolved approach enables signal correction by a simple removal of the parasitic part of reflected photons and by selecting the one associated to the medium by time gating. For example, for these suspensions of kieselguhr particles, in the presence of a reflection peak at 18 ns, the signal selection was carried out by summing the number N of photons arriving between 12 and 16 nanoseconds after the trigger pulse. We note that no continuous wave optical turbidimeter could differentiate the reflection from the scattered light. Whilst providing this feature, TROT will also have a good sensibility for concentrations below 0.01 g/L.

**Figure 9:**
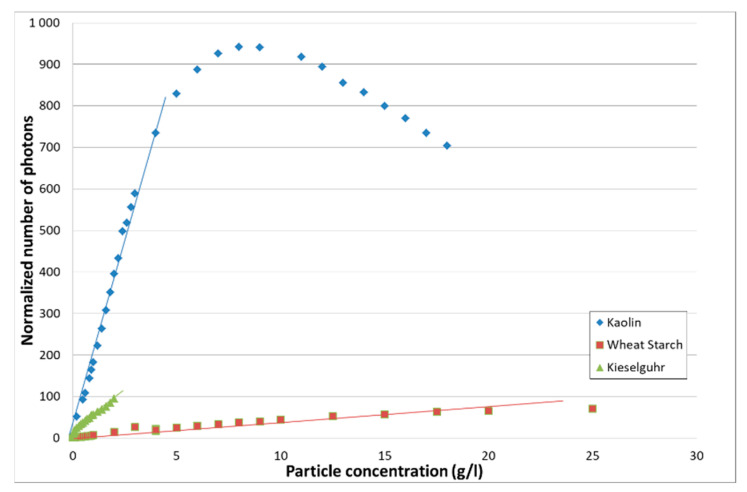
Evolution of the normalized number of photons of TROT as a function of the particle type and concentration.

For the different kinds of particles, the evolution of the total number of collected photons over the acquisition time as a function of the particle concentration is given in Figure 9. Their number was normalized by the number of photons observed for clear water. The normalized number of photons corresponds to the ratio of the number of photons contained in the histogram for a given particle to concentration by the number of photons observed for clear water.

At first approximation, the normalized number of photons has a linear dependance with particle concentration. When applicable, a linear regression analysis was performed according to *N = a_T_ M + b_T_*. The results are given in Table 5.

The normalized number of photons shows a linear dependence on the particle concentration for kieselguhr and wheat starch over the whole explored concentration domain. For kaolin, a maximum is reached after which the normalized number of photons decreases with increasing concentration.

This behavior of TROT for kaolin reflects a high sensitivity to diffusion. It is known that the smaller the particle, the more important the diffusion is.

Table 5 summarizes the results obtained for TROT. As expected for an optical method, TROT is adapted to very low concentrations (~ 0.01 g/L) for common particle sizes. The linearity between the normalized number of photons and the concentration lasts longer than in the case of classical optical turbidity. Even for fine particles such as kaolin, the linearity domain is doubled compared to optical turbidity. For larger particles, the linearity domain reaches the one obtained by acoustics.

## 4. Discussion

If we summarize the observations of the different techniques, we notice that:

- Optical turbidity is well adapted for particle concentration monitoring in the range 0 to 2 g/L. However, it is also clear that, depending on the particle type, optical turbidity can rapidly reach a dysfunctional zone in which the correspondence between the measured turbidity and particle concentration can no longer be established [29].
- For all particles, acoustic attenuation and backscattering show a linear behavior with particle concentration from around 0.1 g/L to several dozens of grams per liter. The concentration range is much larger than the one observed for optical turbidity: acoustic monitoring can reach high concentration values, increasing by at least a factor 4 the values reached by optical turbidity. However, a major disadvantage is that acoustics are not adapted for low concentrations.
- The first results observed for TROT are promising and show that a simple data analysis of the TPSF can be used to monitor particle concentration. A linear relationship between particle concentration and the normalized number of photons of the TPSF is observed for standard concentrations. However, for small particles as kaolin, a decrease in the normalized number of photons with increasing concentration is observed and causes ambiguity on the corresponding concentration value. More sophisticated data analyses, including the use of the photon arrival time might remove the ambiguity and could confirm the potential of the method at high concentrations.

**Figure 10:**
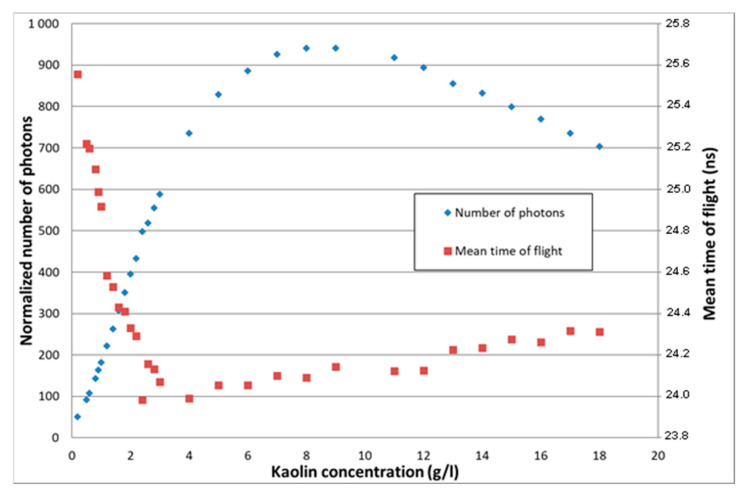
Combined evolution of the normalized number of photons and mean time of flight as a function of the particle concentration.

As suggested, concerning this additional TROT data treatment, Figure 10 shows for example the combined evolution of the normalized number of photons and their mean time of flight as a function of the kaolin concentration. For a given normalized number of photons associated to two kaolin concentrations, the different values of time of flight enable concentration disambiguation. However, an adapted data treatment algorithm still has to be elaborated.

On our laboratory prototype, we showed a clear advantage of time resolved optical turbidity measurements. TROT has at least a similar sensitivity compared to optical turbidity at low particle concentrations where acoustic measurements are inefficient. Alternatively, larger concentration values can be observed by TROT compared to optical turbidity. For high concentrations, the range of TROT is comparable or larger than the one observable by acoustics. More investigations are being made to optimize the multiple parameters of the method (wavelength, observation angle, data treatment etc.,).

## 5. Conclusions

A new optical turbidimeter principle, using time resolved optical tomography, (TROT), is presented and is compared to the performance of a standard broad range optical turbidimeter and to the acoustic backscattering measurements of a standard ADVP.

Both optical methods, commercial optical turbidity and TROT are efficient at low concentrations and have an excellent sensitivity to small particles [30]. Contrariwise, for heavy loaded flows, erroneous behavior of optical turbidity was observed. The optical turbidity decreases with concentration after a certain value depending on the instrument and the type of particle. Depending on the type of turbidimeter, this decrease may occur after a long saturation domain for which the measured optical turbidity value remains at a constant maximal value that is the upper limit of the turbidimeter.

We showed that TROT does not have the same concentration limitations as optical turbidity. The concentration domain is easily extended by a factor of 3 with rough data analysis. The combined response of photon rate and mean time of flight can probably allow a much larger extension. Moreover, the delivered signal is also robust and allows for easy discrimination between parasitic and useful signals.

The time resolved optical turbidity enables satisfactory concentration monitoring on the standard environmental concentration domain. We showed that the number of collected photons is linearly linked to the concentration of suspended particles. The many ways of possible enhancement of the data treatment tools such as using the mean time of flight of the photons or media absorption and diffusion characteristics suggest a very promising method.

More investigations are being conducted to optimize TROT. We are also trying to determine how TROT would react to known optical method biases such as the presence of biofilm or the presence of micro-bubbles [31]. Nevertheless, the application of TROT to the survey of particle load in water showed satisfactory results. On a single modal particle suspension, its performance exceeds the one of standard optical and acoustic backscattering. As such, on known suspensions, TROT might overcome the recently developed instrumentation combining acoustical and optical information as described by Agrawal et al. [32] or Lin et al. [27].

## Figures and Tables

**Table 1:** Parameter description.

Symbol	Parameter
*k_t_*	Acquisition system constant
*ψ*	Near field correction
*M*	Particle concentration
*α_w_*	Water absorption attenuation
*α_p_*	Particle scattering attenuation
*χ_m_*	Normalized total scattering cross-section
*k_p_*	Particle backscattering properties
*ρ_p_*	Particle density
*<a_p_>*	Mean particle radius

**Table 2:** Linear regression analysis of optical turbidity: *T = aM + b*.

Particle Type	Concentration (g/L)	Linear Regression Coefficients		
		*a*	*b*	r^2^ (%)
Kaolin	0.01–2.0	1172.3	−77.026	98.2
Wheat starch	0.01–2.0	419.87	−4.3133	99.96
Kieselguhr	0.01–2.0	316.58	−4.0787	99.74

**Table 3:** Linear regression analysis for slope: *δ = a_s_ M + b_s_*.

Particle Type	Concentration (g/L)	Linear Regression Coefficients		
		*a_s_*	*b_s_*	r^2^ (%)
Kaolin	0.05–9	1.6511	1.9067	96.83
Wheat starch	0.1–42.5	0.6035	2.2864	96.83
Kieselguhr	0.08–2	2.3567	2.108	94.78

**Table 4:** Linear regression analysis for intercept: *γ = a_i_ ln M + b_i_*.

Particle Type	Concentration (g/L)	Linear Regression Coefficients		
		*a_i_*	*b_i_*	r^2^ (%)
Kaolin	0.05–9	0.4575	−11.4530	98.17
Wheat starch	0.1–42.5	0.4524	−11.5660	98.9
Kieselguhr	0.08–2	0.5413	−11.0960	99.85

**Table 5:** Linear regression analysis for the normalized number of photons: *N = a_T_ M + b_T_*.

Particle Type	Concentration (g/L)	Linear Regression Coefficients		
		*a_T_*	*b_T_*	r^2^ (%)
Kaolin	0.01–4	175.77	−11.9340	95.95
Wheat starch	0.01–42.5	3.2415	5.3237	93.82
Kieselguhr	0.01–2	42.392	11.552	97.59

## Data Availability

The data that support the findings of this study are available on request.
